# Impacts of supplementing growing rabbit diets with whey powder and citric acid on growth performance, nutrient digestibility, meat and bone analysis, and gut health

**DOI:** 10.1186/s13568-018-0617-0

**Published:** 2018-05-23

**Authors:** Asmaa T. Y. Kishawy, Shimaa A. Amer, Ali Osman, Shafika A. M. Elsayed, Mohamed E. Abd El-Hack, Ayman A. Swelum, Hani Ba-Awadh, Islam M. Saadeldin

**Affiliations:** 1 Department of Nutrition & Clinical Nutrition, Faculty of Veterinary Medicine, Zagazig University, Zagazig, 44511 Egypt; 2 Department of Biochemistry, Faculty of Agriculture, Zagazig University, Zagazig, 44511 Egypt; 3 Department of Histology and Cytology, Faculty of Veterinary Medicine, Zagazig University, Zagazig, 44511 Egypt; 4 Department of Poultry, Faculty of Agriculture, Zagazig University, Zagazig, 44511 Egypt; 5 Department of Animal Production, College of Food and Agriculture Sciences, King Saud University, P.O. Box 2460, Riyadh, 11451 Saudi Arabia; 6 Department of Theriogenology, Faculty of Veterinary Medicine, Zagazig University, Zagazig, 44511 Egypt; 7 Department of Physiology, Faculty of Veterinary Medicine, Zagazig University, Zagazig, 44511 Egypt

**Keywords:** Rabbit, Whey, Citric acid, Growth performance, Digestibility, Intestinal histology

## Abstract

The present study evaluated the impact of supplementing the rabbit diet with graded levels of whey powder and citric acid. The dietary treatments were as follows: T1, control diet (basal diet); T2, basal diet + 10 g/kg citric acid; T3, T2 + whey powder (7.5 g/kg); T4, T2 + whey powder (15 g/kg); and T5, T2 + whey powder (22.5 g/kg). Results, the T5 diet resulted in the best (P < 0.05) final body weight, body weight gain, feed conversion ratio, protein efficiency, relative growth rate, and dressed weight. The best (P < 0.05) digestion coefficients were associated with the T4 and T5 diets. Rabbits fed diets supplemented with citric acid alone or with addition of graded levels of whey powder showed significantly lower (P < 0.05) intestinal pH than those fed the T1 diet. The T4 and T5 diets resulted in greater CP and ash in the thigh muscle compared with the T1 and T2 diets. Calcium content in the femur bone was higher (P < 0.05) in the T5 group followed by T4 and T3. The wall of different parts of the small intestine improved in the T4 and T5 groups, showing the greatest increase in the small intestinal villi, intestinal glands, and amount of goblet cells. In conclusion, addition of whey powder (1.5, and 2.25%) increased the growth performance, nutrient digestibility and crude protein content of the thigh muscle, and improved the gut health of growing rabbits and the best level was 2.25% whey powder. Citric acid addition had no positive effect on growth performance, nutrient digestibility, crude protein content of the thigh muscle, and the gut health.

## Introduction

Shortage of feed materials with high nutritive value for animal feed has motivated most nutritionists to search for new low-cost and nutritious materials. Whey is a by-product of cheese manufacturing that has high nutritional value and could be used in animal feeding (Rastad 2008). Whey protein is of high biological value containing a reasonable amount of essential amino acids, which rapidly increase plasma amino acids level to improve protein function in the body (Hayes and Cribb 2008). In particular, whey protein contains branched-chain amino acids, especially leucine, which act as modulators in muscle protein metabolism and as key regulators in the initiation translation pathway of muscle protein (Norton and Layman 2006). Whey powder contains approximately 65% lactose that will enhance the growth of lactic acid bacteria (natural probiotics) in the intestinal tract of the animal. In addition, the presence of β-lacto globulin and α-lactalbumin in whey proteins can enhance the immune system of animals and prolong their survival (Shariatmadari and Forbes 2005). Whey protein concentrate has a growth-promoting action in broiler chicks and induces favorable changes in intestinal tract metabolism (Szczurek et al. 2013). Whey powder also contains some traces of organic acids such as lactic acid and citric acid (Tsakali 2010).

Organic acids as citric acid exert antimicrobial activity against rabbit enteropathogenic *E. coli*, (which is natural inhabitant in rabbit intestinal tract and one of enteritis causes) in vitro investigation (Skřivanová and Marounek 2007). Citric acid supplementation in rabbit feed was shown to improve nutrient digestibility and consequently animal performance (Debi et al. 2010), and can also improve salt deposition in the bone, especially calcium, by improving bone metabolism (Dixon and Perkins 1952). Citric acid was shown to have a stimulating effect on intestinal mucosa growth that improves nutrient digestion and animal performance (Romero et al. 2011). Our hypothesis supposed that the combination of whey powder and citric acid would be more effective than their single use. Therefore, this study was performed to investigate the effect of whey powder and citric acid supplementation to growing rabbit diets on growth performance, nutrient digestibility, gut health, chemical composition of the thigh muscle and bone, and muscle calcium and phosphorus contents.

## Materials and methods

### Preparation of whey powder

Whey prepared from cow milk was obtained from the Department of Food Science, Faculty of Agriculture, Zagazig University, Egypt by rennet coagulation according to the methods of (Ha et al. 2014). The whey was pasteurized at 73 °C for 15 s, cooled, lyophilized, and stored in non humid place until use. Liquid whey contained 6.2% dry matter, then samples of whey powder chemically analyzed according to (AOAC 2000).guidelines which give CP 13.2%, EE 2.1%, lactose 67.7%, ash 10.6 and calcium 4.5% and phosphorus 5.6%.

### Animals, diets, and experimental design

The present study was performed at Rabbit Research Unit, Faculty of Veterinary Medicine, Zagazig University, Egypt. All experimental procedures were performed with reference to the Committee of Local Experimental Animal Care and approved by the Nutrition and Clinical Nutrition Department institutional ethics committee at the Veterinary Medicine College, University of Zagazig, Egypt.

One hundred and twenty-five weanlings New Zealand White (NZW) male rabbits with an average body weight of 950 ± 20 g were randomly assigned to five groups with five replicates each (five rabbits/replicate). Rabbits were submitted to a 7-day adaptation period before beginning the trial. Whey was included in rabbit diets at three levels (0.75, 1.5, and 2.25%) with the addition of citric acid (10 g/kg diet). The five dietary treatments were as follows: T1, basal diet without additives (control diet); T2, basal diet supplemented with citric acid (10 g/kg diet) and no whey; T3, T2 + 0.75% whey powder; T4, T2 + 1.5% whey powder; and T5, T2 + 2.25% whey powder. The experiment lasted for 8 weeks. Fresh water was always available to the rabbits. Animals were reared in individual cages under typical management, hygiene, and environmental conditions throughout the experimental period. The basal diet and other experimental diets was formulated in pelleted form and shown in (Table 1), diets formulated according to rabbit (NRC 1977). The proximate chemical analysis of the used feedstuffs and the experimental diets was carried out according to the standard procedures of the (AOAC 2002).

**Table 1:** Chemical composition of the experimental diets (g/kg)

Ingredients	T1	T2	T3	T4	T5
Berseem hay	340	340	340	340	340
Yellow corn	150	150	150	150	150
Barely grain	150	150	142.5	135	127.5
Whey powder	0	0	7.5	15	22.5
Wheat bran	205	205	208	208	208
Soy bean meal 44% CP	139	139	139	139	139
Calcium carbonate	3	3	0	0	0
Premix^a^	3	3	3	3	5
Salt	5	5	5	5	3
Antitoxins (TOXY-NILL plus)	3	3	3	3	3
Anti-coccidial (AMPROL 128)	2	2	2	2	2
Calculated composition(g/kg)^b^					
Digestible energy (DE), kcal/kg	2515	2515	2517	2512	2507
Crude protein	166.8	166.8	167.4	167.5	167.7
Crude fiber	125.2	125.2	125.1	124.7	124.3
Ether extract	26.6	26.6	26.7	26.7	26.8
Calcium	6.29	6.29	8.61	11.99	15.36
Available phosphorus	5.14	5.14	5.15	5.13	5.11
Lysine	9.06	9.06	9.04	9.01	8.97
Cystine	3.04	3.04	3.03	3.02	3.00
Methionine	2.19	2.19	2.18	2.17	2.16
Threonine	5.90	5.90	5.91	5.91	5.90

^a^Premix: each 5 kg contain vit. A (1800,000 IU), vit. D3 (3,000,000 IU), vit. E (3200 mg), vit. B1 (200 mg), vit. B2 (600 mg), vit. B6 (200 mg), vit. B12 (2 mg), calcium antothenate (2000 mg), nicotinic acid (4400 mg), choline (10,000 mg), Fe (10,000 mg), Mn (10,000 mg), Cu (3000 mg), I (200 mg), Co (20 mg), Se (20 mg) and Zn (12,000 mg), Magnesium (100 g)

^b^According to (NRC 1977)

### Growth performance

Body weights (BW) were recorded at the beginning and at the end of the experiment, and feed consumption was measured weekly. The body weight gain (BWG) was calculated as W2 − W1, where W1 is the initial live weight (g) and W2 is the final live weight (g). Feed intake (FI) was recorded weekly. The feed conversion ratio (FCR) was estimated as the amount of feed consumed (g)/BWG (g).The relative growth rate (RGR) was calculated using the following equation (Brody 1945):

$${\text{RGR}} = {\text{W}}2 - {\text{W}}11/2({\text{W}}1 + {\text{W}}2) \times 100$$

Protein efficiency ratio (PER) was determined as the number of grams of weight gain produced per unit of weight of dietary protein consumed (Mcdonald 1966):

$${\text{PER}} = {\text{Live}}\;{\text{weight}}\;{\text{gain}}\;({\text{g}})/{\text{protein}}\;{\text{intake}}\;({\text{g}})$$

### Digestibility

At the end of the experiment, 10 rabbits per group were randomly selected for digestibility trials using the single-cage method. The amount of feed intake and feces excreted were recorded daily. Proximate chemical analysis of feed and feces were determined according to (AOAC 2002) guidelines.

### Carcass traits measurements

At the end of the experimental period, five male rabbits from each treatment group were randomly selected and fasted for 12 h with free water, and then slaughtered. The carcass traits were measured and samples from the small intestine, thigh meat, and bone were taken.

### Intestinal content and muscle pH

#### pH of the duodenum and jejunum contents

Immediately after slaughter, the rabbits were eviscerated, the intestine was removed, and the intestinal contents of the duodenum and jejunum were evacuated into a clean dry porcelain dish; the pH was recorded with a numerical pH meter.

#### pH of the breast muscles

The meat pH was measured at 24 h after slaughter. In brief, 1 g of breast muscle was cooled at 4 °C for 24 h and then placed in a clean dry porcelain dish with distilled water. The muscle was squeezed with a glass rod and then the pH was recorded using the numerical pH meter.

### Chemical analysis of the muscle

Samples from the thigh muscle were taken from the slaughtered animals for analysis of dry matter (DM), crude protein (CP), ether extract (EE) and ash percentages, according to (AOAC 2002).

### Analysis of calcium and phosphorus contents of the meat and bone

Calcium and phosphorus contents of the thigh and femur bone were estimated by an atomic absorption device according to previously described methods (Perkin 1964, Markert 1991). A 1-g sample of bone or meat was combusted in a muffle furnace at 500 °C for 2 days for ash measurements, followed by the addition of 3 ml of nitric acid and 2 ml of perchloric acid. The mixture was left overnight at room temperature and then placed in a hot water bath at 72 °C for 3 h, followed by filtration with Whatman paper; the filtrate was completed to 25 cm with deionized water. Aliquots of the filtrates were used to estimate the concentration of various metals using an atomic absorption spectrophotometer (model 210 VGP, Buck Scientific USD) with an oxidizing air acetylene flame.

### Histological examination

Specimens from the small intestine were collected from all of the rabbit necropsies fixed in 10% neutral buffered formalin. The fixed specimens were dehydrated in an ascending grade of ethanol, cleared in benzene, and embedded in paraffin. Sections (5–7-µm thick) were prepared and stained with hematoxylin and eosin (H & E) according to standard methods (Bancroft 2008). The micro photographs were taken using a digital Dsc-W 130 super-steady cyber shot camera (Sony, Japan) connected to an Olympus BX 21 light microscope.

### Statistical analysis

All data are expressed as the mean ± SD. All data were verified for normal random distribution after transformation (arcsine). One-way analysis of variance was used to determine the effects of different levels of whey with citric acid on growth performance, carcass traits, digestibility, intestinal and muscle pH, chemical analysis of the muscle, calcium and phosphorus contents of the muscle and bone using SPSS version 17 for Windows (SPSS, Inc., Chicago, IL, USA). The differences among means were determined using the post hoc Tukey test. Statements of statistical significance are based on P ≤ 0.05 unless otherwise stated.

## Results

### Growth performance

Table 2 shows the overall impacts of dietary treatments on the growth performance of growing rabbits. The results clearly showed that rabbits fed diets enriched with citric acid plus whey powder (T4, and T5) had improved (P < 0.05) final BW, BWG, FCR, PER, and RGR as compared to those fed the T1 and T2 control diets. This improvement was also greater with increasing levels whey powder from 7.5 to 22.5 g/kg diet. In particular, offering the T5 diet (basal diet + citric acid + 22.5 g whey/kg diet) resulted in the best (P < 0.05) values of all growth performance parameters, excluding FI. Feed intake was not significantly different (P > 0.05) among any of the experimental groups. Diet supplemented with citric acid (1%) alone didn’t cause significant difference in BW, BWG, FCR, PER, and RGR as compared with the control group.

**Table 2:** The effect of graded levels of whey with addition of citric acid on growth performance

Parameters	T1	T2	T3	T4	T5	*P* value
Initial wt (kg)	0.968 ± 0.009	0.952 ± 0.009	0.954 ± 0.015	0.951 ± 0.005	0.955 ± 0.007	0.691
Final BW (kg)	1.955 ± 0.046^c^	2.009 ± 0.058^c^	2.099 ± 0.038^bc^	2.218 ± 0.078^b^	2.401 ± 0.065^a^	≤ 0.001
BWG (kg)	0.987 ± 0.049^c^	1.057 ± 0.060^c^	1.145 ± 0.032^bc^	1.267 ± 0.079^b^	1.447 ± 0.062^a^	≤ 0.001
FI (kg)	4.373 ± 0.66	4.445 ± 0.102	4.448 ± 0.123	4.527 ± 0.181	4.219 ± 0.074	0.437
FCR	4.48 ± 0.249^a^	4.25 ± 0.200^ab^	3.89 ± 0.044^bc^	3.60 ± 0.166^c^	2.95 ± 0.180^d^	≤ 0.001
PER	1.358 ± 0.087^c^	1.424 ± 0.067^bc^	1.539 ± 0.017^bc^	1.671 ± 0.082^b^	2.052 ± 0.115^a^	≤ 0.001
RGR (%)	67.394 ± 2.349^d^	71.197 ± 2.766^cd^	74.959 ± 1.365^bc^	79.678 ± 3.040^ab^	86.035 ± 2.065^a^	≤ 0.001

T1, basal diet with no additives; T2, basal diet supplemented with citric acid (10 g/kg) and no whey; T3, T2 + whey powder (7.5 g/kg); T4, T2 + whey powder(15 g/kg); T5, T2 + whey powder (22.5 g/kg)

*BW* body weight; *BWG* body weight gain; *FI* feed intake; *FCR* feed conversion ratio; *PER* protein efficiency ratio; *RGR* relative growth rate

^a–d^Means within the same row carrying different superscripts are significantly different at (P ≤ 0.05)

### Carcass traits

As shown in Table 3, no significant differences between all experimental groups (P < 0.05) in dressed %, liver %, heart % and kidney %.

**Table 3:** The effect of graded levels of whey with addition of citric acid on carcass traits % relative to life body weight

Relative weights (%)	T1	T2	T3	T4	T5	P value
Dressed	50.98 ± 0.56	54.385 ± 1.07	53.574 ± 0.94	53.705 ± 0.92	54.901 ± 1.62	0.138
Liver	3.206 ± 0.34	3.572 ± 0.215	3.454 ± 0 .29	3.466 ± 0.18	3.682 ± 0.22	0.752
Kidney	0.542 ± 0.03	0.497 ± 0.015	0.548 ± 0.03	0.530 ± 0.02	0.534 ± 0.03	0.665
Heart	0.367 ± 0.05	0 .276 ± 0.046	0.376 ± 0.06	0.363 ± 0.05	0.308 ± 0.04	0.547

T1, basal diet with no additives; T2, basal diet supplemented with citric acid (10 g/kg) and no whey; T3, T2 + whey powder (7.5 g/kg); T4, T2 + whey powder(15 g/kg); T5, T2 + whey powder (22.5 g/kg)

### Digestion coefficient (DC)

The impacts of dietary treatments on the DC of all nutrients studied were summarized in Table 4. In general, there were improvements in the DC of dry matter within all whey supplemented groups in comparison with the T1 diet. Notably, the best (P < 0.05) DC values of crude protein, fat and ash were associated with the T4 and T5 diet. DC of nitrogen free extract was not significant (P > 0.05) between control group and experimental treatments. Addition of citric acid alone (T2) had no significant effect (P > 0.05) on nutrient digestibility.

**Table 4:** The effect of graded levels of whey with addition of citric acid on digestion coefficient (DC) of nutrients (%) on dry matter basis

DC	T1	T2	T3	T4	T5	P-value
DM	73.70 ± 0.21^c^	75.34 ± 0.33^bc^	77.06 ± 1.67^b^	80.72 ± 0.66^a^	82.31 ± 0.86^a^	≤ 0.001
CP	58.49 ± 0.50^b^	58.57 ± 0.84^b^	62.36 ± 3.11^b^	71.40 ± 1.28^a^	76.18 ± 0.63^a^	≤ 0.001
CF	22.78 ± 2.47^c^	27.59 ± 1.06^bc^	31.47 ± 1.88^b^	42.40 ± 2.40^a^	45.51 ± 1.76^a^	≤ 0.001
Ash	51.07 ± 1.02^c^	58.78 ± 0.96^b^	59.72 ± 1.91^b^	70.01 ± 1.53^a^	70.95 ± 2.01^a^	≤ 0.001
EE	52.11 ± 1.92^b^	55.00 ± 1.83^b^	60.22 ± 4.57^ab^	66.97 ± 2.69^a^	68.45 ± 1.39^a^	0.006
NFE	49.77 ± 2.71	53.33 ± 0.18	54.27 ± 5.59	56.94 ± 1.32	62.37 ± 2.32	0.118

T1, basal diet with no additives; T2, basal diet supplemented with citric acid (10 g/kg) and no whey; T3, T2 + whey powder (7.5 g/kg); T4, T2 + whey powder(15 g/kg); T5, T2 + whey powder (22.5 g/kg)

*DM* dry matter; *CP* crude protein; *CF* crude fiber; *Ash* ash; *EE* ether extract; *NFE* nitrogen free extract

^a–c^Means within the same row carrying different superscripts are significantly different at (P ≤ 0.05)

### Intestinal and muscle pH

Addition of 1% citric acid to diets of T2, T3, T4 and T5 had significantly lowered (P < 0.05) intestinal pH values than those fed the T1 diet (Table 5), as the lowest pH values was recorded for T5 group. Muscle pH was not significantly different among all experimental groups.

**Table 5:** The effect of graded levels of whey with addition of citric acid on intestinal and muscle PH, and femur bone analysis on fresh basis

Parameters	T1	T2	T3	T4	T5	P value
Intestinal pH	8.15 ± 0.25^a^	7.36 ± 0.13^b^	7.68 ± 0.15^b^	7.38 ± 0.17^b^	6.66 ± 0.18^c^	≤ 0.001
Muscle pH	6.88 ± 0.12	7.13 ± 0.09	7.04 ± 0.07	7.07 ± 0.07	7.12 ± 0.11	0.357
Femur bone analysis (%)						
Ash	38.08 ± 1.81	38.91 ± 2.15	39.11 ± 0.78	37.83 ± 0.93	39.37 ± 0.45	0.916
Ca	3.12 ± 0.12^c^	3.25 ± 0.22^c^	4.32 ± 0.08^b^	4.88 ± 0.19^ab^	5.05 ± 0.28^a^	≤ 0.001
P	2.90 ± 0.16^b^	3.15 ± 0.25^b^	3.82 ± 0.07^a^	4.03 ± 0.13^a^	4.20 ± 0.17^a^	≤ 0.001

T1, basal diet with no additives; T2, basal diet supplemented with citric acid (10 g/kg) and no whey; T3, T2 + whey powder (7.5 g/kg); T4, T2 + whey powder(15 g/kg); T5, T2 + whey powder (22.5 g/kg)

^a–c^Means within the same row carrying different superscripts are significantly different at (P ≤ 0.05)

### Chemical analysis of the muscle

Excluding crude protein and ash, none of the determined nutrients significantly differed among the dietary treatment groups (Table 6). Rabbits fed the T4, and T5 diets had higher (P < 0.05) CP and ash in their thigh muscle compared with those fed the T1 and T2 diets. No significant differences were found between the T4 and T5 groups for CP, and among the T3, T4, and T5 groups for ash.

**Table 6:** The effect of graded levels of whey with addition of citric acid on chemical analysis of thigh muscle on fresh basis

Parameters (%)	T1	T2	T3	T4	T5	P value
DM	29.27 ± 0.19	29.41 ± 0.24	29.83 ± 0.17	29.79 ± 0.33	30.07 ± 0.41	0.324
CP	21.56 ± 0.05^b^	21.62 ± 0.301^b^	22.23 ± 0.18^ab^	22.39 ± 0.23^a^	22.78 ± 0.30^a^	0.017
EE	2.65 ± 0.01	2.66 ± 0.03	2.70 ± 0.04	2.70 ± 0.07	2.71 ± 0.04	0.754
Ash	0.75 ± 0.01^b^	0.79 ± 0.02^ab^	0.81 ± 0.01^a^	0.81 ± 0.01^a^	0.81 ± 0.02^a^	0.045
Ca*	0.03 ± 0.00	0.03 ± 0.00	0.03 ± 0.00	0.03 ± 0.00	0.03 ± 0.00	0.150
P*	0.28 ± 0.01	0.30 ± 0.01	0.32 ± 0.02	0.33 ± 0.01	0.33 ± 0.02	0.184

T1, basal diet with no additives; T2, basal diet supplemented with citric acid (10 g/kg) and no whey; T3, T2 + whey powder (7.5 g/kg); T4, T2 + whey powder(15 g/kg); T5, T2 + whey powder (22.5 g/kg)

* On dry matter basis

^a,b^Means within the same row carrying different superscripts are significantly different at (P ≤ 0.05)

### Calcium and phosphorus contents in the bone

Table 5 highlights the impact of the dietary treatments on the ash, calcium, and phosphorus contents in the femur bone on a fresh basis. Calcium content in the femur bone was highest (P < 0.05) in the T5 group, followed by T4 and then T3. The phosphorus content of the femur bone was increased (P < 0.05) in the T3, T4, and T5 groups (without significant differences among them) in comparison to the negative and positive controls (T1 and T2). The T1 and T2 diets had similar impacts on calcium and phosphorus contents of the femur bone. The dietary treatments did not have an influence on the ash percentage of the bone.

### Histological examination of the small intestine

**Fig. 1:**
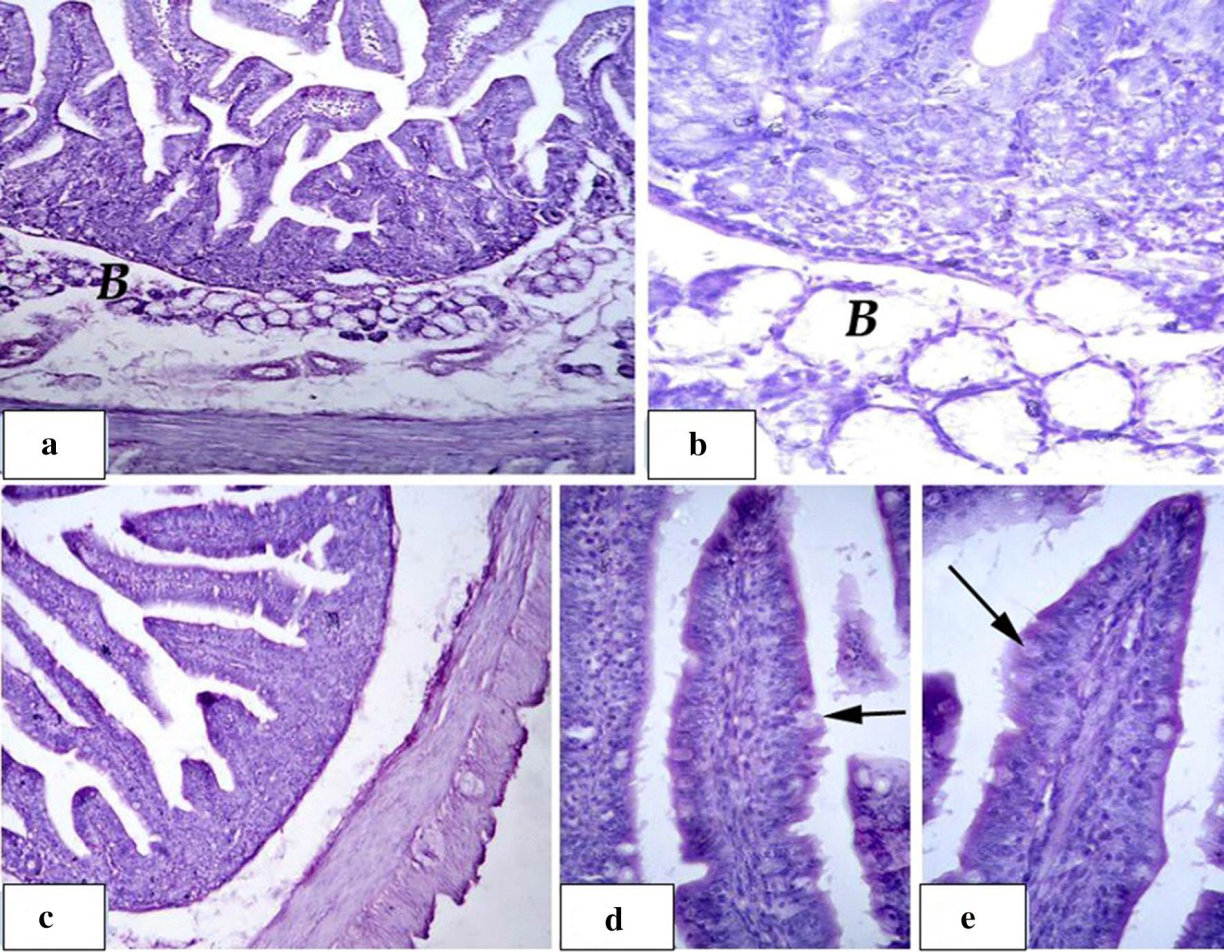
Group T3: **a**, **b** showing normal duodenum mucosa and submucosa with brunners gland (B). **c**–**e** Normal jejunum mucosa and submucosa with normal covering epithelium of columnar absorptive and goblet cells (arrow)

**Fig. 2:**
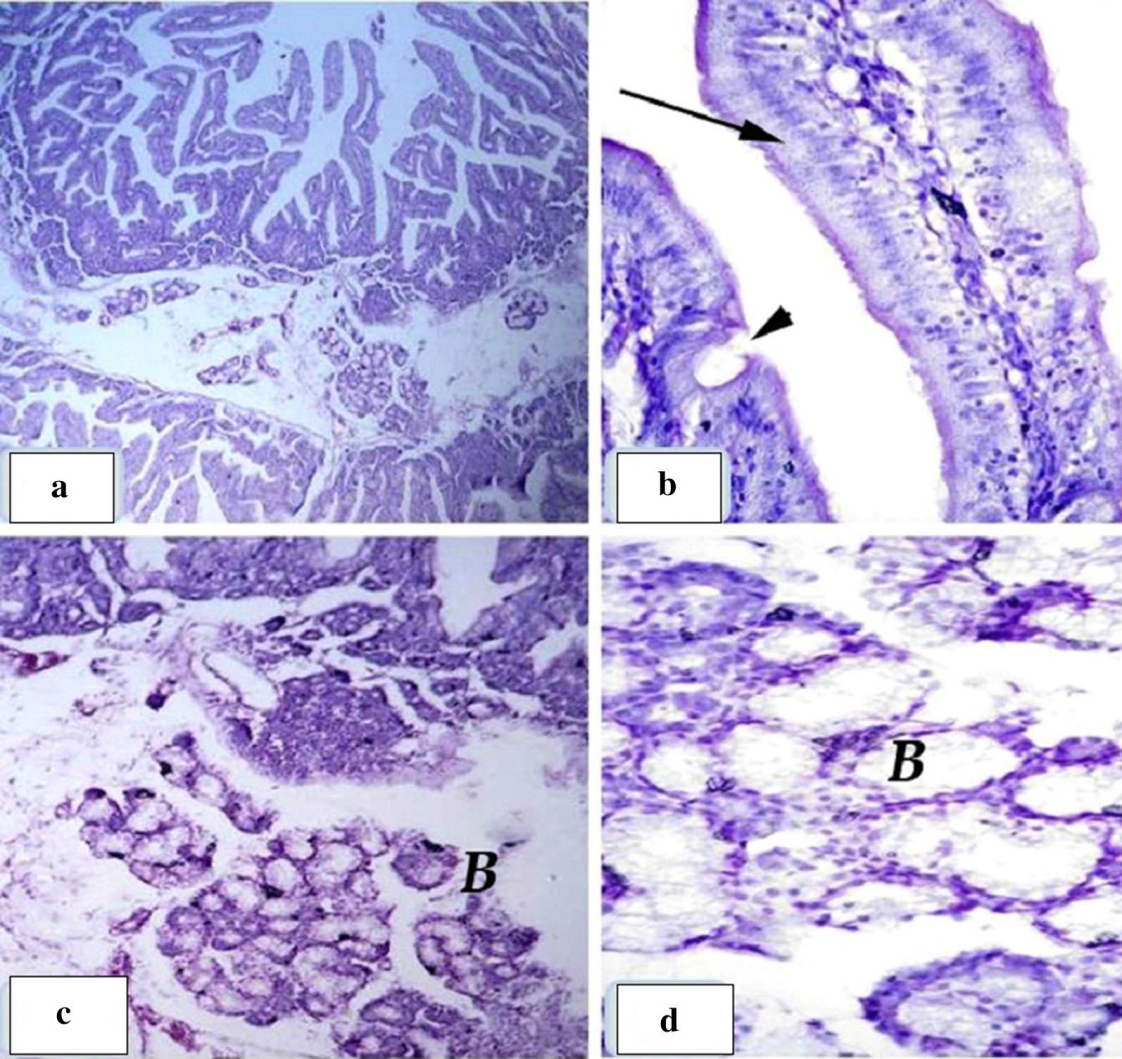
Group T4: **a** showing normal duodenum wall. **b** Normal covering epithelium of columnar absorptive (arrow) and goblet cells (arrow head). **c**, **d** Normal brunners gland (B)

The histological examination of the small intestine of the five dietary groups revealed that groups T1 and T2 exhibited a normal intestinal wall. Groups T3 and T4 showed slight improvement in the intestinal tissue architectures for the mucosa and submucosa (Figs. 1 and 2).

**Fig. 3:**
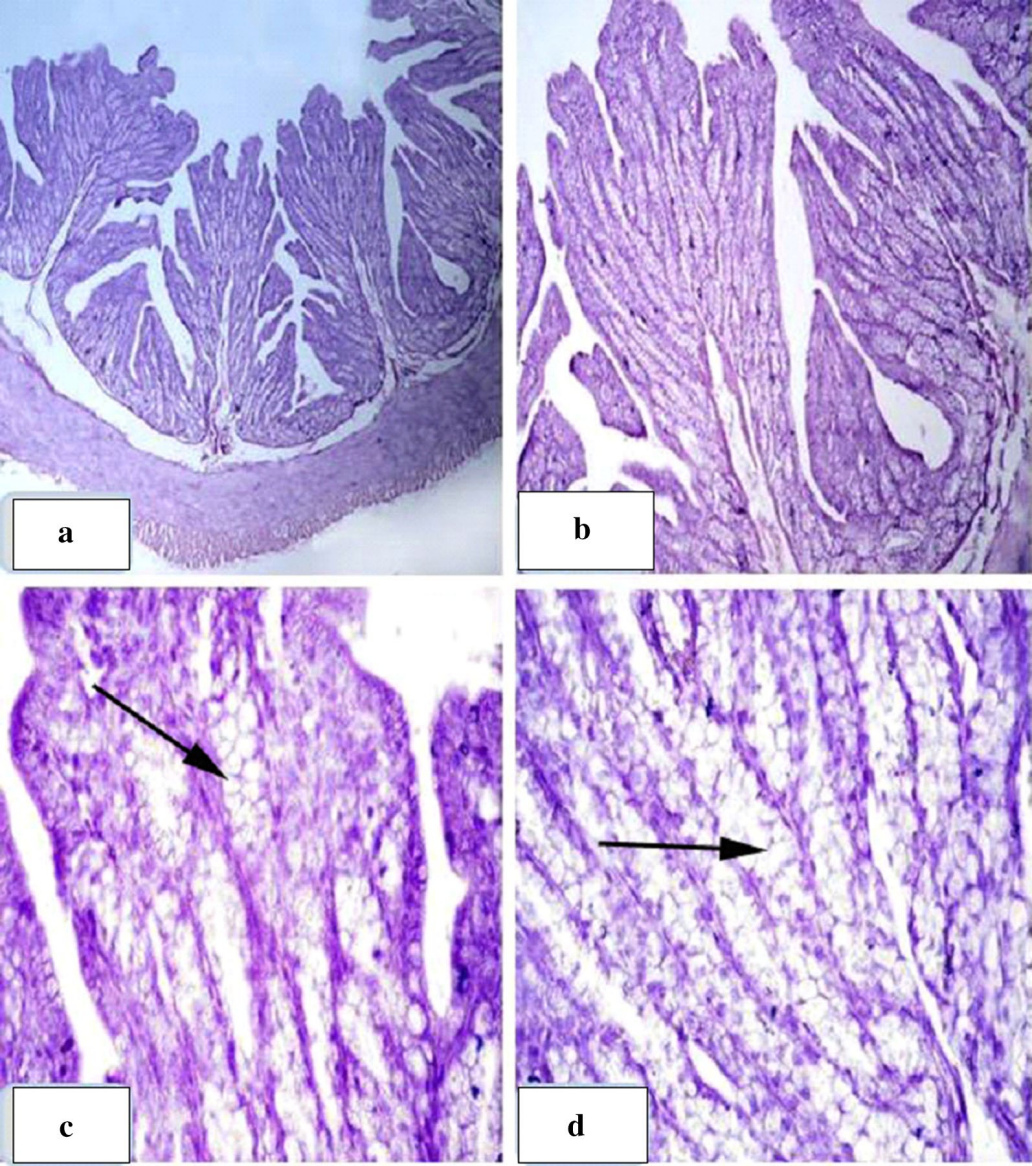
Group T5: **a**, **b** showing normal, intact jejunum mucosa. **c**, **d** Well-developed intestinal gland in the mucosa with increasing number of goblet cells (arrow)

**Fig. 4:**
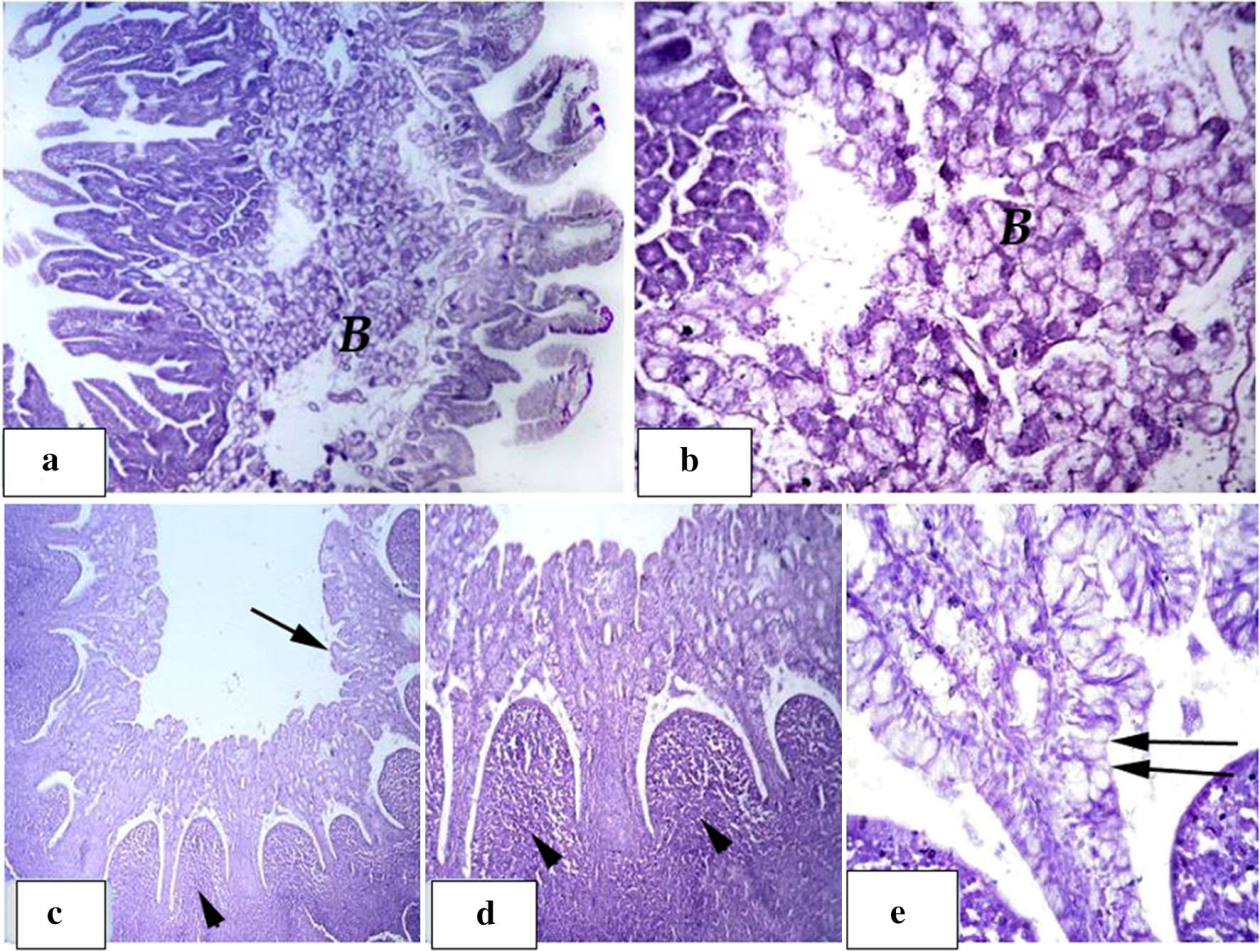
Group T5: **a** showing significant development in duodenum wall. **b** Showing massive layer of mixed brunners gland filling almost of the submucosa. **c**–**e** normal, regular and intact lymphatic nodules; peyers patches within the ileum submucosa (arrow head), intact villi (arrow) with increase in goblet lining cells (double arrows)

Group T5 showed significant improvement in the intestinal wall where regular and intact mucosa were observed with increasing numbers of goblet lining cells, along with obvious mucous secretions on the surface epithelial cells and crypt of Lieberkuhn (Fig. 3). In the duodenum, the Brunner’s glands were well-developed and appeared to be formed from mixed acini with serous and mucous granules. Regular and intact lymphatic nodules, Peyer’s patches, were observed in the ileum (Fig. 4).

## Discussion

The availability of low-cost raw materials for animal feed with high feed efficiency is a major target of nutritionists and animal producers. Whey is a by-product of the cheese industry, which is economical and has a high nutritive value (Fallah 2016). There are limited researches on application of whey on rabbit diets. Our results revealed that addition of citric acid (1%) to the basal diet didn’t cause significant improvement in the growth performance compared with the control group. Addition of whey powder (1.5, and 2.25%) to the basal diet along with citric acid led to significant improvement in BW, BWG, FCR, PER and RGR and the level 2.25% whey powder is better than 1.5%. This improvement in growth performance may be attributed to significant increase in the digestibility of crude protein, fat, crude fiber as well as well-developed intestinal glands and an increase in the number of goblet cells in groups T4 and T5 due to the higher contents of whey, which contain peptides and amino acids. Gilbert et al. (2008) reported that nutrient digestibility in broiler chicken improved because of an increase in the number of peptides, amino acids, and glucose transporters, as well as promoted enzyme production. These findings are in agreement with the data of (Fallah 2016), who reported that supplementation of a chicken diet with different levels of dried whey and protexin probiotic improved the body weight of chickens at 42 days. A similar result was also obtained by Majewska et al. (2009) who found that the addition of undiluted fresh acid whey and lactic acid to drinking liquid twice a week for 4 h improved broiler chicken performance. Malik et al. (2015) further supported these results in that the addition of whey protein at 2, 4, and 6% to broiler feed was found to improve their FCR. These consistent improvements in FCR, body weight, and total gain may be related to the higher digestibility of whey protein and reasonable essential amino acid content of whey protein (Hoffman and Falvo 2004).

Debi et al. (2010) showed that providing growing rabbit’s rations with 0.5, 1, 1.5, 2, and 2.5% citric acid improved the DM, CP, and EE digestibility but did not affect CF and NFE. This is consistent with data of Hoffman and Falvo (2004), who reported that whey protein powder had a high amount of essential amino acids such as leucine and cystine, which could improve protein digestibility and bioavailability.

Biggs and Parsons (2008) showed reduced growth performance at 21 days of chick growth as a result of addition of citric acid 4% whereas no significant effect by 3% citric acid addition In contrast, Romero et al. (2011), Uddin et al. (2014), reported improved growth performance due to addition of citric acid to rabbit feed. (Kliševičiūtė et al. 2016), reported that inclusion of butyric acid and organic acid salts such as citric acid and propionate increased the body weight and weight gain, and improved the FCR of rabbits. Citric acid creates an acidic environment in the intestinal tract that prevents the growth of pathogenic bacteria, allowing the nutrient to become more available to the animal, which contributes to the observed improvement in animal performance (Abdel-Fattah et al. 2008).

The dressed carcass percent, liver, heart, and kidney were not affected by experimental treatments. In contrast, Bahari et al. (2015) demonstrated that the addition of 4% whey powder to the broiler ration improved carcass weight, carcass percent, breast weight, drum stick weight, and wings weight.

The results of our study showed that feeding the T2, T3, T4, and T5 diets lowered the pH of the duodenal content compared to that of the T1 group, and the T5 group had the lowest pH of the duodenal content overall. Similarly, Kliševičiūtė et al. (2016) found that the addition of butyric acid and organic acid salts lowered the pH of the duodenal and ileum contents. Moreover, Uddin et al. (2014) reported that inclusion of 1% citric acid lowered the small intestine pH, although the effect was not statistically significant. Moreover, Szczurek et al. (2013) showed that whey protein concentrate lowered the cecal content pH and intestinal content pH. This effect is considered to be related to the activity of the intestinal microflora of the rabbit intestine, including lactic acid bacteria and coliform bacilli, which can ferment lactose sugar, a component of whey powder, and produce volatile fatty acids in the intestinal lumen, which would decrease the pH of cecal and intestinal contents. In the present study, the thigh muscle pH was not affected by any of the diet treatments after 24 h within the range data reported by Majewska et al. (2009), who found that the pH value of the breast muscle after 24 or 48 h was not affected by providing fresh undiluted whey in liquid form to broiler chicks.

The results showed that T4 and T5 improved the CP content of the thigh muscle of rabbits, which is related to the high levels of essential amino acids and branched-chain amino acids in whey powder such as leucine Hoffman and Falvo (2004). In addition, whey protein has sufficient amounts of cystine, which stimulates glutathione levels that enhance the antioxidant activity of the body (Bounous 2000) along with immunity (Ha and Zemel 2003). Whey inclusion at 0.75, 1.5, and 2.25% significantly increased the calcium and phosphorus contents of the bone, but did not affect bone ash contents. These results can be clarified in consideration of the findings of Dixon and Perkins (1952), who suggested that a local increase in citric acid in the bone matrix due to co-precipitation during bone salt composition. Thus, we speculate that the high bioavailability of calcium and phosphorus contents of whey powder was the main cause for the high calcium and phosphorus retention in the bone.

From the obtained results we can conclude that addition of whey powder (1.5, and 2.25%) increased the growth performance, nutrient digestibility and crude protein content of the thigh muscle, and improved the gut health of growing rabbits and the best level was 2.25% whey powder. Citric acid addition had no positive effect on growth performance, nutrient digestibility, crude protein content of the thigh muscle, and the gut health.

## Abbreviations

NZW: New Zealand White
BW: body weights
BWG: body weight gain
FCR: feed conversion ratio
PER: protein efficiency ratio
DM: dry matter
CP: crude protein
EE: ether extract
NFE: nitrogen free extract
DC: digestion coefficient
